# Myeloid-derived suppressor cells and the pathogenesis of human immunodeficiency virus infection

**DOI:** 10.1098/rsob.210216

**Published:** 2021-11-10

**Authors:** Mahmoud Mohammad Yaseen, Nizar Mohammad Abuharfeil, Homa Darmani

**Affiliations:** Department of Biotechnology and Genetic Engineering, Faculty of Science and Arts, Jordan University of Science and Technology, Irbid 22110, Jordan

**Keywords:** tat protein, gp120, PD-1/PD-l1, regulatory T (treg) cells, microbial translocation, T-cell dysfunction

## Abstract

There are several mechanisms by which human immunodeficiency virus (HIV) can mediate immune dysfunction and exhaustion during the course of infection. Chronic immune activation, after HIV infection, seems to be a key driving force of such unwanted consequences, which in turn worsens the pathological status. In such cases, the immune system is programmed to initiate responses that counteract unwanted immune activation, for example through the expansion of myeloid-derived suppressor cells (MDSCs). Although the expansion of immune suppressor cells in the setting of systemic chronic immune activation, in theory, is expected to contain immune activation, HIV infection is still associated with a remarkably high level of biomarkers of immune activation. Paradoxically, the expansion of immune suppressor cells during HIV infection can suppress potent anti-viral immune responses, which in turn contribute to viral persistence and disease progression. This indicates that HIV hijacks not only immune activation but also the immune regulatory responses to its advantage. In this work, we aim to pave the way to comprehend how such unwanted expansion of MDSCs could participate in the pathology of acute/primary and chronic HIV infection in humans, as well as simian immunodeficiency virus infection in rhesus macaques, according to the available literature.

## Graphical abstract

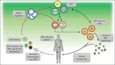

## 1. Introduction

Both arms of the innate immune system—namely the cellular (monocytes/macrophages (Mo/M*Φ*), dendritic cells (DC), natural killer (NK) cells, basophils/mast cells, polymorphonuclear neutrophils (PMN), eosinophils and the newly identified innate-like cells including B1 and marginal zone B cells) and humoral (complement system) responses—play indispensable roles in: (i) recognizing/sensing and clearance of ‘cell-debris, foreign substances, and invading pathogens'; (ii) antigen internalization and presentation to shape the adaptive immune system activation; (iii) immune-activated downregulation (the so-called immune regulation) upon the clearance of pathogens and abnormal cells or during chronic immune activation; (iv) immune tolerance; (v) maintaining the architecture of tissues (so-called tissue remodelling); as well as (vi) newly emerging functions, in particular, immunological memory after re-infection [1–7]. Innate immune cells can sense both pathogen-associated molecular patterns (PAMPs) expressed by pathogenic and nonpathogenic microbes, and/or danger-associated molecular patterns (DAMPs) exhibited in stressed or damaged cells/tissues by different classes of receptors, the so-called pattern recognition receptors (PRRs) [8]. To our knowledge, PRRs can be categorized into five major classes of receptors: (i) toll-like receptors (TLRs), (ii) nucleotide-binding oligomerization domain (NOD)-like receptors (NLRs), (iii) C-type lectin receptors, (iv) retinoic acid-inducible gene (RIG)-I-like receptors (RLRs), and the recently identified class of PRRs, namely (v) absent in melanoma 2 (AIM2)-like receptors (ALRs) [9–11]. Interaction of PRRs of innate immune cells with PAMPs or DAMPs can initiate immune responses by activating specific signal-transduction pathways which depend on the pattern of host–pathogen interaction. This, in turn, results in activation of specific transcription factors (e.g. nuclear factor kappa-light-chain-enhancer of activated B cells (NF-κB), activator protein-1 ‘AP-1’, mitogen-activated protein kinase, cyclic adenosine monophosphate ‘cAMP’-response element-binding protein, CCAAT-enhancer-binding proteins (c/EBP) and interferon regulatory factors, etc.). Transcription factor activation results in the release of different cytokines such as interleukin (IL)-1, IL-6, tumour-necrosis factor (TNF)-α and interferon (IFN)-γ, as well as chemokines, enzymes, adhesion molecules and regulatory factors, among others [1,2,12–15], all of which are essential for driving the inflammatory process. In the context of infections, if the innate immune system failed to eliminate or contain the infectious pathogen, it activates a much more specific branch of the immune system, namely the adaptive immune system. This highlights the fact that the innate immune system is crucial for the primary and secondary immune responses [1,2]. It is vital to remember that the subsequent adaptive immune responses of both the cellular (T-cell responses) and humoral (antibodies generated by plasma cells) arms are primarily polarized and shaped according to the innate immune system stimulus [2]. However, this process is governed by different factors, including (i) the nature of the invading pathogen(s); (ii) the type of innate immune cells (e.g. DC, M*Φ* or PMN etc.) encountered; (iii) the anatomical site and the microenvironment where pathogenic invasion occurs (e.g. blood circulation, lymphatic and non-lymphatic tissues); as well as (iv) the pattern(s) of pathogen–host interaction(s) [1,2].

In fact, clearance of the pathogen(s) and infected cells is considered to be a major goal of immune system activation upon infection. However, certain pathogens such as the ‘human immunodeficiency virus' (HIV) can successfully evade both the innate and adaptive immune responses and persist in infected hosts [2]. Upon HIV infection, immune responses become chronically activated and inflammation biomarkers become measurable almost at any time point during the course of infection. The latter is, however, considered to be an unwanted consequence. This is because of the fact that keeping immune responses continuously activated (uncontrolled), even at low levels, could (i) worsen the immunopathology of such infections (especially those that thrive under inflammatory conditions, including HIV infection [16]), (ii) lead to certain pathological conditions that are not directly related to the invading pathogen(s) (i.e. tissue damage; depending on the site of inflammation), and (iii) result in immune dysfunction and exhaustion, all of which contribute to disease progression, one way or another [1,2]. This underscores the importance of activating the regulatory arm of the immune system to prevent the unwanted consequences of chronic immune activation. Theoretically, we can say that the immune regulation process plays an indispensable role in terminating the inflammatory processes, especially in infectious agents that mediate chronic immune activation. However, this does not mean that we should exclude the role in the settings of acute immune activation, since most subsequent immune responses are shaped according to the acute immune responses [1,2]. Nevertheless, the question arises as to whether this is true when talking about HIV infection. The answer to this question will be addressed in the following discussion.

Immune regulation is a highly sophisticated process that is orchestrated by different immune suppressor cell populations. Under normal physiological conditions, different immune cell populations that belong to the innate immune system (e.g. innate-suppressor cells of myeloid origin called myeloid-derived suppressor cells (MDSCs)) and/or the adaptive immune system (adaptive-suppressor cells including regulatory T (Treg) cells and regulatory B (Breg) cells) work together in harmony to terminate inflammation and revert back to the homeostatic state [6,7,17–19]. On the other hand, it is essential to keep in mind that although immunosuppressive cells could have a positive impact in the context of containing inflammation [20], especially if this is accomplished as soon as they become activated, failure to achieve such a goal could result in unwanted consequences, as seen in chronic inflammatory pathological conditions, such as observed in different types of cancer, certain autoimmune disorders and various infections [6,20]. In other words, prolonged activation of immune regulatory processes (i.e. chronic activation of immunoregulatory cells) results in the suppression of specific immune responses to a given immunopathological condition, thereby leading to disease persistence/progression (discussed below) [6,7].

In this paper, we will focus our discussion on the role of innate immune suppressor cells, namely MDSCs, in the pathogenesis of HIV infection alone. Therefore, the role of MDSCs in HIV patients co-infected with other pathogens such as cytomegalovirus, hepatitis C virus (HCV) or *Mycobacterium tuberculosis*, or suffering from other inflammatory pathological conditions, including cancer, will not be included in this work. It is important to note that understanding the biological properties of MDSCs in normal and abnormal conditions is necessary to understand the pathophysiology of human disease, and subsequently to develop new potential therapeutic strategies [6,7]. Hence, in order to clearly understand the following discussion of the role of MDSCs in HIV infection, we encourage readers to examine our recently published review articles on this topic [6,7].

Let us begin by reviewing the general properties of MDSCs. As potent immunoregulatory cells of myeloid origin (i.e. express myeloid markers), MDSCs comprise a mixture of mature and immature immune suppressor cell populations that mainly express either monocytic or granulocytic features. As such, there are two main populations of MDSCs, namely monocytic-MDSCs (M-MDSCs) and granulocytic-MDSCs (G-MDSCs or PMN-MDSCs). Under normal physiological conditions, MDSCs are expanded as a result of inflammatory immune responses for the purpose of terminating the damaging effect of unwanted inflammatory responses and/or achieving immune tolerance, as seen during pregnancy and lactation [7]. On the other hand, under non-physiological pathological conditions, including cancer and infectious diseases, such as HCV and influenza virus infection, expansion of such cells could worsen the clinical status of these pathological conditions [6,7]. The role of MDSCs during HIV infection will be addressed in the following discussion.

## 2. The role of myeloid-derived suppressor cells in human immunodeficiency virus infection

HIV is a splendid example of a chronic infectious pathogen that successfully evades and invades the immune system and mediates chronic immune activation [1,2]. Apart from the direct immunopathological effects mediated by the virus itself, chronic immune activation, upon HIV infection, leads to immune dysfunction and exhaustion, both of which contribute to disease progression and HIV persistence [1,2,21–23]. To counteract such chronic immune activation, the expansion of immunoregulatory cells during HIV infection is mediated [24,25]. It is essential to keep in mind that although immunoregulatory responses could play a role in preventing/limiting the cell/tissue damage mediated by chronic immune activation upon HIV infection, these immune responses could limit anti-HIV immune responses resulting in defective immune responses against this virus [24–28], and thus enhancing its persistence, as aforementioned. From this point of view, in the following sections, we will focus on the expansion of MDSCs and their pathological roles during the primary and chronic phases of HIV infection.

### 2.1. Myeloid-derived suppressor cells expansion during the course of human immunodeficiency virus and simian immunodeficiency virus infections

As previously stated, it is widely accepted that MDSCs are expanded in various pathological conditions including cancer, sepsis, autoimmune disorders, microbial infections and allergy, among others. This expansion is associated with suppressive immune responses that worsen the status of these pathological conditions, as detailed in our recently published reviews [6,7].

The story of MDSCs in HIV infection began when the following scenario was presented. During the course of HIV infection, most patients can partially control viremia for a certain time. Unfortunately, after a short time, this control is lost in most cases. To address whether MDSCs, as a potent immunosuppressive cell population, play a role in this event during HIV infection, in 2012 and for the first time in humans, Vollbrecht *et al*. [29] reported a significant increase in the frequency of MDSCs, particularly PMN-MDSCs, in chronically HIV-infected patients when compared to healthy subjects. They were also the first group to describe the positive association between the expansion of MDSCs and HIV disease progression. Indeed, this observation was documented upon comparing two HIV-infected populations in different clinical stages, namely HIV progressors (i.e. HIV patients with a viral load greater than 5 × 10^4^ copies per ml and CD4^+^ T-cell counts lower than 250 cells per µL) and HIV controllers (i.e. HIV patients with a viral load lower than 5 × 10^3^ copies per µl, and CD4^+^ T-cell counts greater than 5 × 10^2^ per µl, without highly active antiretroviral therapy (ART) ‘i.e. HAART-naive HIV patients’). They showed that HIV controllers had a significantly lower frequency of MDSCs. Of particular importance, they also reported that MDSCs, particularly PMN-MDSCs, are elevated in chronically HIV-infected individuals that were HAART-naive, when compared with HIV patients that were on HAART with undetectable viral loads, as well as healthy control subjects. Interestingly, the levels of PMN-MDSCs rapidly plunged after six weeks of HAART initiation. Initially, these results could lead to a postulation that the driving force of such elevation in MDSC counts, during chronic HIV infection, is viral replication. On the contrary, M-MDSCs were shown to have no impact on HIV disease progression in the study of Vollbrecht *et al*. [29], because no differences in their frequencies were observed in the different clinical stages of HIV infection. Furthermore, Vollbrecht *et al*. [29] demonstrated that there was a negative correlation between the levels of PMN-MDSCs and CD4^+^ T-cell counts. These findings suggest that MDSC expansion is associated with disease progression markers during HIV infection.

Shortly thereafter, several groups of researchers also confirmed that MDSCs are expanded during the chronic phase of HIV infection. In 2013, Qin *et al*. [30] observed that M-MDSCs, but not PMN-MDSCs, are dramatically elevated in seropositive chronic HIV-infected patients when compared to healthy subjects. One could ask why there is such difference in MDSC phenotypes, even within the same pathological condition; HIV infection in our case? The answer to this question lies in the fact that MDSCs are very sensitive to some manipulations, including the methods of sample processing. For example, cryopreservation can reduce the viability of MDSCs, in particular the PMN-MDSCs [7]. Therefore, it is rational to link this difference in MDSC phenotypes to the differences in sample processing between the two studies, especially since Qin *et al*. used cryopreserved samples. However, according to Qin *et al*., this is not the case because they repeated their experiments on the same HIV patients using fresh samples to exclude the possibility of sample processing being the underlying cause of such differences in MDSC phenotypes observed in their study and that of the Vollbrecht group [29]. Qin *et al*. suggested that this could be due to the difference in the systemic cytokine responses, as a consequence of the difference in HIV strains between patients. Another plausible explanation for this difference is the clinical stage of HIV infection. Generally, it is agreed that the inflammatory microenvironment plays a critical role in driving the differentiation and expansion of one MDSC population over the other [6,7]. In the context of HIV infection, as the clinical stage of HIV infection advances, immune depletion (mainly CD4^+^ T cells) and alteration in the inflammatory microenvironment become more observable. As such, we could refer the expansion of M-MDSCs over PMN-MDSCs in Qin group's study, at least in part, to the clinical stage of enrolled patients. This is true, especially taking into account that HIV patients in the study of Vollbrecht *et al*. [29] were at a less advanced clinical stage than those in the study of Qin *et al*. In other words, most of the enrolled HIV patients (approx. 70%) in the study of Qin *et al*. were at the C3 clinical stage according to the USA CDC classification system (i.e. patients exhibited acquired immunodeficiency syndrome (AIDS) symptoms; CD4^+^ T cells greater than 200 per ml). However, other plausible explanations could also exist and will be discussed later. With respect to the impact of ART on the frequency of MDSCs, they have shown that despite the fact that the frequency of M-MDSCs were decreased after the initiation of HAART to a level below the baseline level, still the levels of M-MDSCs were significantly higher in HIV-infected patients compared to those in healthy subjects. This may indicate that even prolonged HAART fails to renormalize the levels of M-MDSCs. Their results have also strongly supported the direct correlation between the levels of M-MDSCs, but not PMN-MDSCs, and disease progression since M-MDSCs levels were directly associated with the viral load and indirectly with the CD4^+^ T-cell counts. Meanwhile, there were no differences in the frequency of PMN-MDSCs between HIV-infected patients and healthy individuals. Qin *et al*. also confirmed the direct link between HIV viremia and M-MDSC expansion, since a dramatic decrease in plasma viremia coincided with a notable decrease in MDSC frequency. Furthermore, they showed that HIV-infected patients in advanced disease stages (less than 200 CD4^+^ T cells/µl) and especially those with AIDS symptoms had higher M-MDSC levels than those without AIDS symptoms.

Such inconsistency in results regarding which population of MDSCs (i.e. M-MDSCs or PMN-MDSCs) is expanded during the chronic phase of HIV infection still exists in later studies. For example, Bowers *et al*. [31], Tumino *et al*. [32] and Zhang *et al*. [33] confirmed the results of Vollbrecht *et al*. [29] in that PMN-MDSC populations become expanded over M-MDSC populations during the chronic phase of HIV infection. On the other hand, in accordance with the results of Qin *et al*. [30], Garg *et al*. [34] and Wang *et al*. [35] also demonstrated that M-MDSCs are expanded during chronic HIV infection. However, the study of Garg *et al*. [34] could not determine whether PMN-MDSCs are expanded or not, since the anti-CD14 (a marker for monocytic origin) but not anti-CD15 (a marker for granulocytic origin) antibody was used in their study. Therefore, it is not possible to exclude the likelihood of PMN-MDSC expansion, since the two populations could be equally expanded simultaneously within the same patient, or one population may be predominant over the other [36]. Furthermore, the total number of enrolled HIV patients (i.e. patient sample) in the study of Garg *et al*. [34] was too small, so the interpretation of the results cannot be generalized. However, since the *in vitro* findings that M-MDSCs are expanded upon exposure to infectious and non-infectious HIV particles, as well as upon exposure to gp120, were consistent with the *in vivo* results, this could strengthen the results of this study.

MDSCs are reported to be expanded in chronic inflammatory conditions including cancer [6,7]. The results of initial studies on HIV infections were in agreement with these studies, in that the expansion of MDSCs firstly confirmed during the chronic but not acute phase of HIV infection. However, later on, particularly in 2017, different groups of investigators confirmed that MDSCs are also expanded during the early/acute phase of HIV infection (also known as primary HIV infection) [33,37]. Of these groups, Tumino *et al*. [37] studied the kinetics of MDSC expansion from the acute to the chronic phase of HIV infection and determined the factors involved in such expansion. They compared the frequency of MDSCs in HIV patients in acute and chronic phases with healthy donors and observed that MDSCs, particularly PMN-MDSCs, are expanded very early, within the first weeks (particularly in Fiebig stages II/III) post-HIV infection and remained high over time. Zhang *et al*. [33] also investigated the alterations of MDSCs in primary HIV infection and their association with disease progression. They reported that MDSCs, particularly PMN-MDSCs, are expanded during both the primary and chronic phases of HIV infection when compared to healthy donors, and such expansion was shown to be associated with disease progression markers. Furthermore, according to their results, HIV replication did not seem to be involved in PMN-MDSC expansion during acute HIV infection. This is especially true, since there was no correlation between viral load and PMN-MDSC expansion in acutely infected HIV patients. Other groups, such as Grutzner *et al*. [38] and Agrati *et al*. [39], have also confirmed that MDSCs are expanded during the primary phase of HIV infection.

Using animal models, Dross *et al*. [40] were the first to study the kinetics of MDSC expansion in simian immunodeficiency virus (SIV)-infected rhesus macaques. Similar to what has been observed in the study of Tumino *et al*. [37], MDSC expansion was confirmed in the early phase of SIV infection and such expansion continued to the chronic phase. Moreover, Sui *et al*. [36] were the first to study the expansion and the tissue distribution of MDSCs during SIV infection in rhesus macaques. In agreement with previous results, Sui *et al*. [36] also confirmed that MDSCs are expanded in peripheral blood during the chronic phase of SIV infection. Paradoxically and unexpectedly, they observed that the level of MDSCs was decreased in the bone marrow of chronic SIV-infected rhesus macaques and such decreases were associated with disease progression markers. This observation was of a surprise because of the fact that, in all the previous studies on different inflammatory pathological conditions including cancer, once the expansion of MDSCs occurs, the distribution of such cells was almost consistent in different anatomical compartments including blood circulation and bone marrow, and such expansion was shown to be associated with disease progression [6,7]. It is worth mentioning that the results of Sui *et al*. [36] are very important and highly unexpected, thus, we are planning to address their results in a separated work.

Taken together, these findings clearly emphasize the expansion of MDSCs during the acute and chronic HIV and SIV infections and confirm that such expansion has a negative impact on the disease progression status. Therefore, in the next sections, we will address the pathological role played by MDSCs in a comprehensive manner in the hope of gaining a better understanding of the pathophysiology of HIV infection.

## 3. Myeloid-derived suppressor cell expansion and the pathogenesis of human immunodeficiency virus infection

The mechanisms of immune suppression and the pathological role played by MDSCs during different pathological conditions including cancer are relatively well established when compared to HIV infection. However, in recent years, great achievements in MDSC/HIV research field have been made. As such, in this section, we address this issue in a comprehensive manner and we have collected the available results on MDSC and HIV infection alone during the period from 2012 to the time of paper submission.

### 3.1. Myeloid-derived suppressor cells during chronic human immunodeficiency virus infection

**Figure 1.**
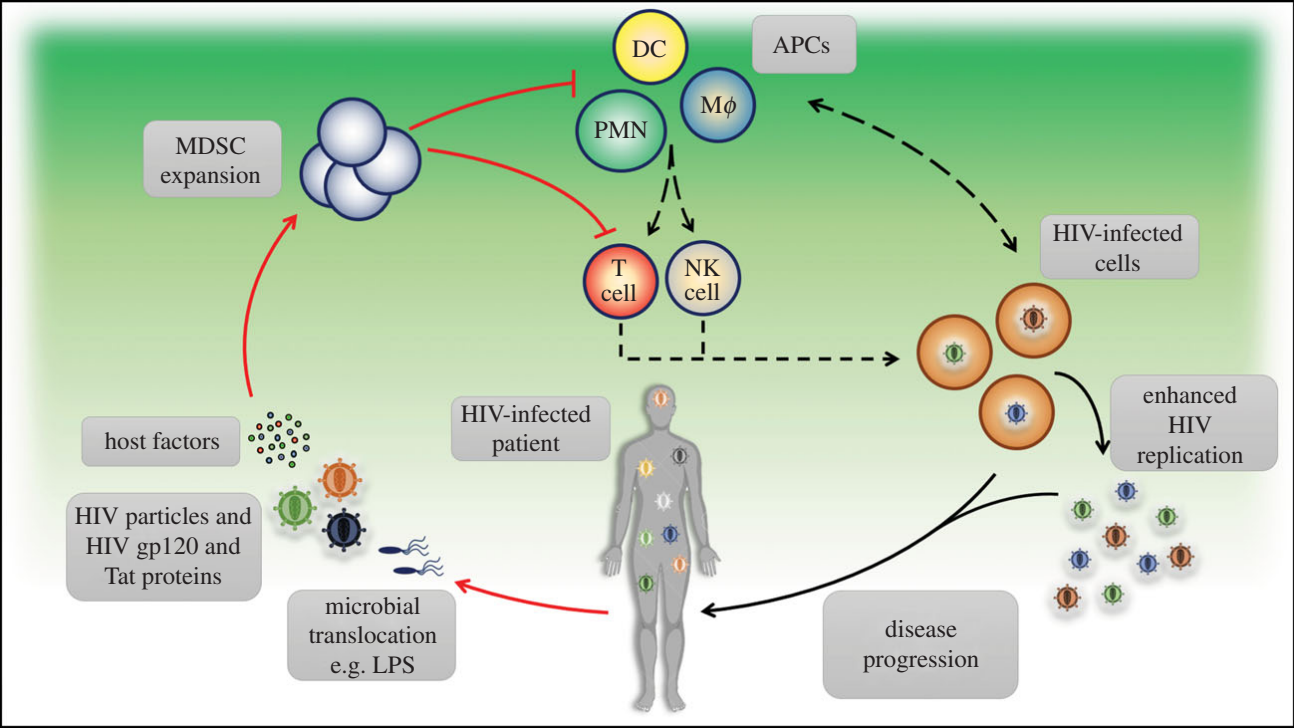
Expansion of MDSCs and the pathogenesis of HIV infection. Upon HIV infection, MDSC expansion is driven by different factors including HIV particles (infectious or non-infectious), HIV proteins (e.g. HIV gp120, Nef and Tat proteins), host factors (e.g. inflammatory cytokines and molecules) and microbial byproducts (e.g. lipopolysaccharide ‘LPS’) upon microbial translocation. These pathologically expanded MDSCs can suppress both the innate (i.e. antigen-presenting cells ‘APC’ such as dendritic cells ‘DC’, macrophages ‘MΦ’, polymorphonuclear neutrophils ‘PMN’ and natural killer ‘NK’ cells) and adaptive immune responses (both the helper and cytotoxic T cells). As a result, immune responses against HIV-infected cells will be restricted, which, in turn, will enhance HIV replication, one way or another, thus entering a vicious cycle leading to disease progression.

Although MDSCs can suppress immune responses mediated by different types of immune cells including DCs, monocytes and NK cells, MDSCs mainly suppress immune responses mediated by T cells [6,7]. This is the main reason why studies on HIV infection focus on the suppressive role of MDSCs on T cells. Vollbrecht *et al*. [29] were the first to demonstrate the pathological role of MDSCs during the chronic phase of HIV infection. With respect to their immunosuppressive function, it is essential to know that MDSCs can mediate their suppressive activity directly via engagement with other immune cells and/or indirectly via mediating the expansion of other immunosuppressor cells such as Tregs, or secreting immunosuppressive molecules (figure 1). To address this issue, Vollbrecht and colleagues incubated PMN-MDSCs with peripheral blood mononuclear cells (PBMC)-obtained from HIV controllers. Interestingly, PMN-MDSCs were able to mediate the expansion of ‘CD4^+^CD25^+^FoxP3^+^’ Tregs. Indeed, this finding was consistent with the significant correlation between the frequency of MDSCs and Tregs in HAART-naive HIV progressors. On the other hand, MDSCs derived from HIV progressors dramatically decreased the proliferative capacity of cytotoxic (CD8^+^) T cells of both healthy and HIV-infected controllers. This was observed after co-culturing of MDSCs derived from HIV-infected progressors with phytohemagglutinin (PHA)-activated CD8^+^ T cells from healthy individuals and MDSCs of HIV-infected controllers with CD8^+^ T cells stimulated with Gag/Nef-peptide which significantly reduced their proliferative capacity when compared to incubation with MDSC-depleted PBMC from HIV-infected progressors, both of which can limit anti-HIV immune responses resulting in virus persistence and consequently disease progression. These data indicate that the direct and indirect immunosuppressive activities of MDSCs are inseparable events, which, in part, reflects the complexity of the immune response network.

To study the suppressive mechanisms of MDSCs derived from HIV-infected patients, Vollbrecht *et al*. [29] have shown that PMN-MDSCs of their cohorts express IL-4 receptor alpha (IL-4R*α*), which paradoxically was shown to be expressed in immunosuppressive M-MDSCs derived from cancer patients with suppressive functions [41]. Initially, this information provided an indication that, during HIV infection, PMN-MDSCs could have suppressive effects on T cells, at least in part through the IL-4R*α* pathway. However, it is important to remember that the primary objective of the study of Vollbrecht *et al*. was to determine whether MDSCs are expanded during chronic HIV infection or not, and, if so, to determine whether such expansion may have a role in HIV disease progression. This explains why this study did not focus on the suppressor mechanisms by which MDSCs could inhibit anti-HIV immune responses.

A year later, Qin *et al*. [30] were the second group to study the impact of MDSC expansion during chronic HIV infection, in a more comprehensive manner. Regarding the mechanism(s) by which M-MDSCs mediate their suppressive effects, Qin *et al*. [30] reported that there was a moderate but significant association between M-MDSC expansion and detection of CD38^+^CD8^+^ T cell subsets, which is known to be associated with disease progression markers (i.e. high viral load, loss of CD4^+^ T cells and abnormal chronic immune activation). In HIV infection, the expansion of CD8^+^ T cells expressing activation markers such as CD38 was shown to be associated with disease progression in primary and chronic HIV-1 infections [42,43]. This suggests that MDSCs could mediate their suppressive activity indirectly, at least in part, via stimulating such an activated/exhausted subset of T cells.

Unlike M-MDSCs from healthy individuals, Qin *et al*. [30] showed that M-MDSCs from HIV patients exhibit suppressive effects on cell proliferation and IFN-γ production for both helper (CD4) and cytotoxic (CD8) T cells in a dose- and contact-dependent manner. Importantly, this supports the idea that MDSCs function differently in pathological settings. Indeed, there are different mechanisms by which M-MDSCs can suppress T-cell responses. _L_-arginine metabolism and its metabolic products are essential for the immune suppression mediated by MDSCs, as reported in non-HIV pathological conditions [44]. As such, Qin *et al*. [30] compared the levels of _L_-arginine metabolic products arginase, nitric oxide (NO) and reactive oxygen species (ROS) in the peripheral blood samples obtained from HIV patients and healthy subjects. Interestingly, a remarkable increase in the activity of arginase was observed in PBMC from HIV patients compared with healthy subjects. This increase in the activity of arginase in M-MDSCs was associated with increased levels of *Arg1* expression (the gene that encodes for arginase; which is an enzyme responsible for arginase 1 ‘ARG1’ activity). By contrast, when comparing the levels of NO in the plasma of HIV patients and healthy subjects, there was a decrease in NO levels in the plasma from HIV patients. This decrease in NO levels was coupled with a decrease in the level of *iNos* expression (the gene that encodes for iNOS enzyme; which is an enzyme responsible for the production of NO). These results indicate that HIV infection could induce the expression of *Arg1* while repressing the expression of *iNos* in M-MDSCs. Lastly, there was no obvious difference in ROS levels between HIV patients and healthy subjects. This indicates that the suppressive effects were due to the induction of Arg1. The study of Qin *et al*. [30] also showed that *in vitro* HIV infection of normal PBMC obtained from healthy individuals led to a remarkable increase in M-MDSCs, indicating that HIV infection contributes directly to the expansion of MDSCs. Furthermore, the administration of recombinant Tat protein to PBMC cultures from healthy subjects resulted in a remarkable increase in MDSC generation, suggesting that Tat protein by itself could play a role in MDSC expansion during HIV infection. This report was the first of its kind to demonstrate that viral proteins, namely HIV Tat protein, can mediate MDSC expansion separately from the viral replication. Perhaps, if they measured the levels of Tat protein and compared them to the frequency of MDSCs in peripheral blood of HIV patients, a stronger correlation can be made, hence we suggest future investigations to clearly determine the impact of Tat protein on the expansion of MDSCs.

Qin *et al*. [30] also reported that M-MDSCs express high levels of HIV co-receptors, namely CCR5 and CXCR4, while expressing lower levels of CD4, and that M-MDSCs have the potential to be directly infected by the virus. Although this possibility exists, the virus seems to have no cytotoxic effects on MDSCs, especially because there is a positive association between MDSC counts and viral load. An additional interesting finding in this study was that MDSCs can also enhance viral replication in CD4^+^ T cells, since there was a significant increase in HIV-1 p24 antigen in supernatants of MDSCs/T-cell co-cultures with high MDSCs/T-cells ratios (1 : 1) compared to the low MDSCs/T-cell ratios (1 : 10). This enhancement was shown to be dependent on direct cell-to-cell contact. Taken together these data show that MDSCs not only participate in limiting specific T-cell anti-viral immune responses and mediating immune exhaustion, but also, they enhance viral replication, all of which can lead to viral persistence and disease progression, suggesting that targeting MDSCs could have a potential clinical implication.

To further understand how HIV dampens anti-HIV immune responses mediated by T cells that, in turn, lead to disease progression, Bowers *et al*. [31] have conducted their investigations on the most abundant leucocyte in the human body, namely the PMN. Although they reported no difference in the phenotype of neutrophils in healthy and HIV-infected patients, there was a significant increase in programmed death ligand-1 (PD-L1) expression on neutrophils of HIV patients, and as such, these PD-L1^high^ neutrophils were described as immunosuppressor cells. In addition, they were isolated from the low-density (LD) gradient (i.e. monocytic fraction) which is consistent with the findings of Cloke *et al*. [45,46]. Furthermore, recent advances in MDSC characterization have revealed that MDSCs comprise a mixture of mature and immature myeloid cells (IMCs) with immunosuppressive function [7], and PMN-MDSCs are also isolated from the LD gradient. Although the exact aetiology of LD neutrophils remains unclear, some investigators believe that neutrophils could acquire this phenotype after degranulation, so that their density becomes similar to that of PBMC and thus may become co-segregated in the LD gradient of the mononuclear cell fraction. Accordingly, the neutrophils in the study by Bowers *et al*. can be described as PMN-MDSC or, at least, as PMN-MDSC-like cells. Bowers *et al*. [31] observed a significant association between PD-L1 expression and the viral load. This is, especially, true because the drop in viral load upon initiation of ART resulted in a significant decrease in the expression of PD-L1 on LD neutrophils. On the other hand, no differences were observed in PD-L1 expression on LD neutrophils between elite HIV-infected controllers (who naturally maintain HIV replication below the limit of detection (50 HIV RNA copies/ml by standard assays) and healthy subjects. This suggests that restoring normal MDSCs could play a role in controlling HIV infection, one way or another.

It goes without saying that the immunoregulatory function of PD-1/PD-L1-axis has become an attractive research area in recent years. In the study of Bowers *et al*. [31], the depletion of PD-L1^high^ LD neutrophils (PMN-MDSC) from PBMC of HIV donors resulted in a significant increase in CD4^+^ and CD8^+^ IFN-γ-producing T cells. A comparable increase in IFN-γ production by T cells was also reported upon specific stimulation of PMN-MDSC-depleted PBMC samples, from HIV patients, with viral antigens (HIV-1 gag polypeptide pool), non-specific stimulation with PHA or anti-CD3/CD28 antibodies. This suggests that the inhibitory function mediated by this immunosuppressive population of neutrophils was independent of specific antigen stimulation. In addition, Bowers *et al*. [31] have shown that the elevation of PD-L1 expression on LD neutrophils is directly associated with T-cell exhaustion and immuno-senescence markers, PD-1 expression on both CD4^+^ and CD8^+^ T cells and CD57 expression on CD4^+^ T cells. Furthermore, the expression level of PD-L1 on LD neutrophils directly correlates with the level of ARG-1 in plasma of HIV patients and indirectly with the expression of CD3ζ chain on T cells. In fact, ARG-1 is known to limit the responsiveness of T cells through downregulating the expression of CD3ζ chain by the depletion of a critical metabolite required for T-cell functions, namely L-arginine [47]. Of note, during antigen stimulation, the interaction of PD-1 expressed on T cells with PD-L1 expressed on different antigen-presenting cells, such as DCs and monocytes/macrophages, is known to suppress T-cell activation mediated by T-cell receptor (TCR).

Exposing DC, monocytes and other immune cells to activated/inactivated HIV particles and activation of TLR-7/8 or type 1 interferon (IFN-α) signalling pathways in MDSCs are known to increase the expression of PD-L1 [48–50]. Consistently, Bowers *et al*. [31] have shown that all of the above-signalling events significantly contribute to the increased expression of PD-L1 on PMN-MDSC. This indicates that the virus itself, as well as certain factors produced by host cells, can positively affect the expression of PD-L1 on LD neutrophils. Other factors such as microbial by-products including lipopolysaccharide (LPS) have been shown to increase the expression of PD-L1 on neutrophils upon *in vitro* exposure [31]. Although the concentration of LPS used in this study was significantly higher than that present in the circulation of HIV patients [51,52], we cannot exclude their partial involvement in mediating PD-L1 expression *in vivo*. An important issue to be mentioned here in this regard is that HIV infection impairs gut-epithelial barrier integrity leading to microbial translocation [1]. LPS is one of the microbial by-products that can be detected in the blood of HIV-infected patients. Bowers *et al*. [31] examined neutrophils in the blood of HIV patients, but not in other compartments, such as lymphatic tissues including gut-associated lymphatic tissues (GALT). It is essential to recognize that during HIV infection, major events (including HIV replication, massive depletion of immune cells and microbial translocation) occur in these compartments because of the presence of a considerable number of target immune cells including T cells, suggesting a need to study such immunological changes in lymphatic tissues including GALT. For more details about the role of neutrophils in gut impairment and microbial translocation during HIV infection, we have recently published a comprehensive review [1]. Overall, these data indicate that the elevation of PD-L1 expression on LD neutrophils during HIV infection is a multifactorial process and participates in immune suppression during HIV infection leading to HIV persistence and disease progression.

Again, downregulating the expression of CD3ζ chain on T cells has been shown to be associated with T-cell hypo-responsiveness to antigen stimulation (i.e. T-cell anergy). This is true, especially, knowing that CD3ζ chain, as a part of TCR, plays indispensable roles in coupling antigen recognition with TCR and subsequent downstream signal-transduction pathways leading to T-cell activation [53–55]. HIV infection is associated with T-cell dysfunction and exhaustion [56]. Thus, it is not surprising to that HIV infection inhibits T-cell function, at least in part, through reducing the expression of CD3ζ chain on both αβ and γδ T cells [57,58], which is, indeed, an unwanted consequence that directly correlates with disease progression markers [59]. There are different factors that can down-modulate the expression of CD3ζ chain on T cells including, but not limited to tryptophan starvation and accumulation of tryptophan catabolites [60], as well as deprivation of the amino acid, L-arginine [61]. Both mechanisms are governed by the activity of indoleamine 2,3-dioxygenase (IDO) and expression of ARG-1, respectively, which are produced by different immune cells including MDSCs [62–64]. To delineate the role of MDSC expansion during HIV infection in this context, Tumino *et al*. [32] have confirmed the results of previous studies which reported a significant expansion in PMN-MDSC population in HIV patients when compared to healthy subjects. Although the previous reports indicated that there is a direct correlation between viral load and MDSC level, Tumino *et al*. [32] did not observe such correlations. In addition, an inverse correlation between CD4^+^ T-cell count and the frequency of MDSCs in HIV patients was reported by different groups; however, Tumino *et al*. [32] did not observe such correlations in PMN-MDSC frequency between HIV patients (different groups) with CD4^+^ T-cell count greater than 400 cells ml^−1^. Still, HIV patients with CD4^+^ T-cell counts below 400 cells ml^−1^ were shown to have a significant difference in PMN-MDSC frequency when compared to other HIV groups with CD4^+^ T-cell counts greater than 400 cells ml^−1^. Importantly, Tumino *et al*. [32] delineated the direct role of PMN-MDSCs in downregulating the expression of CD3ζ chain in different T-cell populations (CD4^+^, CD8^+^, Vγ9 Vδ2T cells). This observation was consistent with the previous observation that HIV infection is associated with a remarkable reduction in CD3ζ chain expression on Vγ9 Vδ2T cells [58]. Interestingly, such roles played by PMN-MDSCs are not only restricted to HIV patients but also observed in cells isolated from healthy donors, suggesting that the effect on the expression of CD3ζ chain is a general feature of PMN-MDSCs. Furthermore, such effects were dependent on direct cell-to-cell contact, as demonstrated in *in vitro* studies. The study of Tumino *et al*. [32] also confirmed that downregulating the expression of CD3ζ chain on T-cells results in their hypo-responsiveness, i.e. reduced IFN-γ production by T cells upon stimulation with specific (HIV-peptides; Gag and Nef) or non-specific (phosphoantigen) antigens. It is important to remember that downregulating the expression of CD3ζ chain on T cells is not an irreversible event. As such, depleting the PMN-MDSCs from T-cell cultures resulted in the restoration of CD3ζ chain expression on T cells and restoration of T-cell function (IFN-γ production). PMN-MDSCs were able to downregulate the expression of CD3ζ chain by interfering with E74-like ETS transcription factor 1 (ELF-1), a transcription factor involved in regulating the expression of CD3ζ molecule [65,66]. Yet, the exact mechanism(s) by which PMN-MDSCs downregulate the expression of CD3ζ chain remains to be determined. However, the amino acid, L-arginine, seems to play a role in this context, especially, because L-arginine starvation is reported to decrease the expression of CD3ζ molecule on Jurkat T cells and such reduction was associated with their limited response to antigen stimulation [67]. Since PMN-MDSCs express high levels of ARG-1, which, in turn, leads to increased activity of arginase and thus L-arginine catabolism and depletion from the microenvironment, we postulate that PMN-MDSCs could mediate the downregulation of CD3ζ chain expression, at least in part, through the expression of ARG-1. Therefore, targeting such immunoregulatory cell populations could be of potential therapeutic implication for controlling HIV infection.

With respect to the already established function of MDSCs in that they inhibit T-cell functions, Garg *et al*. [34] have also shown that coculturing of HIV gp120-expanded CD33^+^ cells with autologous CD4^+^ and CD8^+^ T cells can significantly limit their capacity to produce IFN-γ, when compared to controls (CD33^+^ cells alone). Importantly, such inhibitory function was dependent on direct cell-to-cell contact. As previously mentioned, MDSCs could mediate their suppressive function using the biochemical and metabolic soluble mediators such as ROS, iNOS and ARG-1 [68–70]. In the study of Garg *et al*. [34], the mechanism(s) by which gp120-expanded MDSCs mediated their inhibitory function (i.e. inhibition of IFN-γ production by T cells) was dependent only on iNOS and ROS, since the neutralization of these two, but not ARG-1, upon coculturing of gp120-expanded MDSCs with CD4^+^ or CD8^+^ T cells restored their capacity to produce IFN-γ. Another important soluble molecule that has the capacity to suppress immune responses including those mediated T cells is IL-10 (please refer to our recent review for more details about the immunosuppressive role of MDSCs/IL-10 [6]). IL-10 is produced by a wide range of immune cells including CD4^+^ T cells, particularly Treg cells, and MDSCs [6]. The production of IL-10 is elevated during HIV infection, and such elevation was shown to be directly associated with virus replication [71]. To delineate whether gp120-expanded MDSCs contribute to this elevation in IL-10 production, Garg *et al*. [34] have shown that neither gp120-expanded MDSCs nor CD4^+^ T cells were able to produce IL-10 when cultured alone. Interestingly, coculturing the gp120-expanded MDSCs with CD4^+^ T cells resulted in the release of copious amounts of IL-10, and according to the intracellular staining used to detect IL-10 production, CD4^+^ T cells but not the gp120-expanded MDSCs were identified to be the source of IL-10 production. The last finding in this study was that gp120-expanded MDSCs can mediate the expansion of Treg cells, the primary source of IL-10 in this study. These findings indicate that expanded MDSCs contribute to immune suppression during HIV infection, at least in part, through mediating the expansion of Treg cells. Undoubtedly, using purified and isolated cells does not represent the complexity of the interaction in different cell populations involved in MDSC mediation in the blood of HIV patients, and thus, the experimental system used in this study is considered a limitation. As such, Garg *et al*. [34] suggested to use whole blood PBMC to overcome this limitation in future investigations.

### 3.2. Myeloid-derived suppressor cells during acute human immunodeficiency virus infection

Tumino *et al*. [37] were among the first groups to study the role of MDSCs during acute HIV infection. They did not find any correlation between the frequency of PMN-MDSCs and immune activation (T-cell activation) or inflammatory factors, thereby, ruling out the possibility of immune activation being the driving force that regulates the expansion of MDSCs during the early phase of HIV infection. By contrast, this possibility still exists when discussing MDSC expansion during the chronic phase of HIV infection. In particular, this is because Tumino *et al*. [37] observed a correlation between immune activation markers such as soluble IL-2 receptor (sIL-2R; which is a marker of immune activation and disease progression in HIV-infected individuals [72,73]), interferon alpha (IFN-α) and monocyte chemoattractant protein-1 (MCP-1) among others, and PMN-MDSC frequency in chronic-infected HIV patients. To investigate the soluble factors that could regulate MDSC expansion, a panel of 40 cytokines and growth factors were examined in both acutely and chronically HIV-infected patients [37]. Interestingly, only tumour-necrosis factor-related apoptosis-inducing ligand (TRAIL; which is a multifunctional member of the tumour-necrosis factor cytokine-superfamily, closely related to Fas ligand, and is known to induce apoptosis in several cell types including MDSCs [74–76]) was shown to be correlated with the frequency of PMN-MDSCs in acutely infected HIV patients, especially the Fiebig stages II/III and IV. Although the levels of plasma TRAIL were higher in both groups when compared to those of healthy subjects, there was no difference between acutely and chronically infected HIV patients. Unexpectedly, in contrast with chronic HIV infection, a negative correlation between TRAIL levels and PMN-MDSC frequency was observed in acute phases of HIV infection, suggesting that TRAIL could play a different role in the context of MDSC expansion according to the disease stage. In order to clarify this unexpected observation, they investigated the role of GM-CSF in this context for two reasons. First, according to their results, GM-CSF was the only cytokine it's the level of which was significantly higher in chronically infected HIV patients when compared to acutely infected patients. Second, this cytokine plays a critical role in mediating the expansion of MDSC [62]. As expected, culturing of PBMC from acutely infected patients with GM-CSF and recombinant-TRAIL (r-TRAIL) abrogated TRAIL-mediated apoptosis, explaining the positive correlation between the frequency of PMN-MDSCs and the plasma level of TRAIL during chronic HIV infection. Although Tumino *et al*. [37] observed no correlation between the frequency of PMN-MDSCs and viral load or CD4^+^ T-cell count in both acutely and chronically infected HIV patients, the negative impact of PMN-MDSCs on HIV disease progression cannot be excluded. This is mainly because the CD4^+^ T-cell counts of the enrolled patients (in all groups) in their study was relatively high. However, they provided evidence that the expansion of PMN-MDSCs starts very early during HIV infection, and this expansion continues to the chronic stage of HIV infection. Furthermore, PMN-MDSC expansion during HIV infection is influenced by the clinical stage and the host immune responses including TRAIL and GM-CSF.

Zhang *et al*. [33] also confirmed that MDSCs, PMN-MDSCs in particular, are expanded during the early and late infection stages of HIV. In agreement with previous studies, they have shown that a significant association exists between the level of PMN-MDSCs and disease progression markers. For example, the PMN-MDSC level negatively correlates with CD4^+^ T-cell counts, and positively correlates with viral load and immune activation manifested by inducing CD38^+^CD8^+^ T-cell populations. It must be remembered that the fact that CD38^+^CD8^+^ T cells play a critical role in the pathogenesis of HIV infection has been well established and could also be used as a marker of disease progression in: primary HIV infection; T-cell activation; residual virus replication in chronically HIV-infected patients receiving ART; virological failure in HIV-infected youths and children receiving ART; and HIV dissemination into the central nervous system [42,77–81]. However, a higher level of PMN-MDSCs was observed in HIV patients with lower CD4^+^ T-cell counts during the acute phase of infection when compared to patients with high CD4^+^ T-cell counts, reflecting the importance of CD4^+^ T cells in shaping the clinical status. Additionally, in agreement with previous studies, Zhang *et al*. [33] also confirmed that PMN-MDSCs derived from HIV patients can suppress the proliferation and IFN-γ production of TCR-stimulated CD8^+^ T cells. To determine the mechanism by which PMN-MDSCs could mediate immune suppression during HIV infection, Zhang *et al*. questioned whether PD-L1 and galectin-9 (Gal-9), which are co-inhibitory molecules that play a notable role in immune-related disorders [82], are expressed on MDSCs or not. Consequently, they confirmed a significant increase in the expression of PD-L1 but not Gal-9 on PMN-MDSCs which could be mediated in part through IL-10. Furthermore, they demonstrated the direct association between PD-L1 expression on PMN-MDSCs and the expression of its ligand on CD8^+^ T cells, namely PD-1, during the primary and chronic phases of HIV infection. This indicates that the PD-1/PD-L1 axis is involved in the suppressive function of MDSCs, especially since blocking this axis using a PD-L1 blocking antibody resulted in a notable restoration of CD8^+^ T-cell function (i.e. proliferation capacity and IFN-γ production) in PMN-MDSC-inhibited CD8^+^ T-cells system. These findings suggest that MDSCs could be targeted, at least in part, through the PD-1/PD-L1 axis to restore anti-HIV immune responses mediated by T cells.

**Figure 2.**
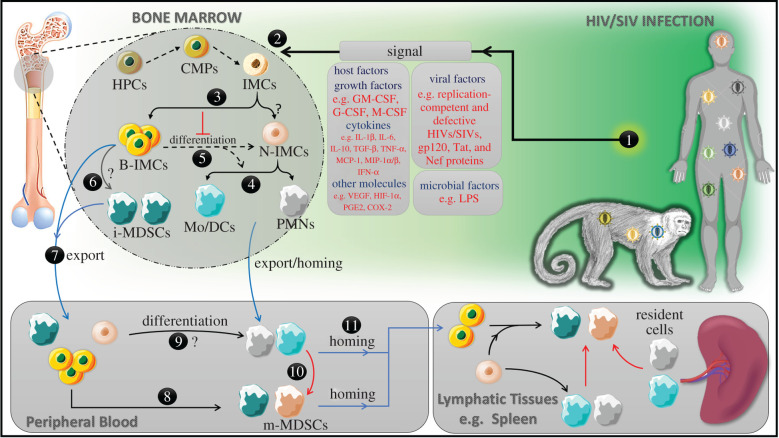
A proposed scenario describing the expansion of MDSCs during HIV infection in humans and SIV infection in rhesus macaques. (1) Replication-competent/defective HIV/SIV particles, HIV gp120, Tat, and Nef proteins, and host factors produced in response to HIV infection, as well as microbial products such as LPS consequent to microbial translocation upon gut-epithelial barrier damage, all of which could spark (2) the initiation of emergency myelopoiesis in which a shift in differentiation to common myeloid progenitors (CMPs) from haematopoietic progenitor cells is induced. Next, IMCs differentiate from CMPs. (3) If IMCs differentiate normally (the so-called normal-IMC (N-IMC)), then they will generate mature agranulocytes (monocytes/dendritic cells ‘Mo/DCs’) and granulocytes (such as polymorphonuclear neutrophils ‘PMNs’, among others) in the bone marrow. (4) Accordingly, mature Mo, DCs and PMN can be released to the peripheral tissues. (5) On the other hand, abnormally activated (i.e. blocked at an immature stage due to the viral and host signals (the so-called blocked-IMC ‘B-IMC’) cannot differentiate into mature Mo/DCs or PMNs. (6) At this stage, B-IMC could become immunosuppressive due to exposure to the inflammatory molecules in the surrounding microenvironment. Accordingly, we called them immature myeloid-derived suppressor cells (immature-MDSCs (i-MDSCs)). (7) As the haematopoietic output increases, it is normally to expect that the exporting rate of both the B-IMCs and N-IMCs outside the bone marrow to peripheral tissues will be increased. Once in the peripheral blood, (8) a similar scenario to what happened to the B-IMCs in the bone marrow could occur in blood circulation. In other words, they may become immunosuppressive (i.e. i-MDSCs) or may be incorporated to the inflammatory locations and secondary lymphatic tissues (11). On the other hand, (9) blood N-IMC could continue the differentiation process into mature cells Mo, DCs and PMN or infiltrated to the inflammation sites and lymphatic tissues (11). It is essential to know that (10) PMNs, Mo and DCs in peripheral blood could be reprogrammed into an immature immunosuppressive state and become i-MDSCs or acquire immunosuppressive activity at their mature state, mature-MDSCs (m-MDSCs). Alternatively, PMNs and Mo/DCs could be attracted to the inflammatory sites where they could become i-MDSCs or m-MDSCs. A similar scenario could also be applied for B-IMC and N-IMC upon infiltration to lymphatic tissues. Importantly, the suppressive activity of the MDSCs (i-MDSCs and m-MDSCs) may be increased at the inflammatory sites, since the exposure time to inflammatory molecules is increased. Of note, the red arrows refer to reprogramming, while the blue arrows refer to export/homing.

**Figure 3.**
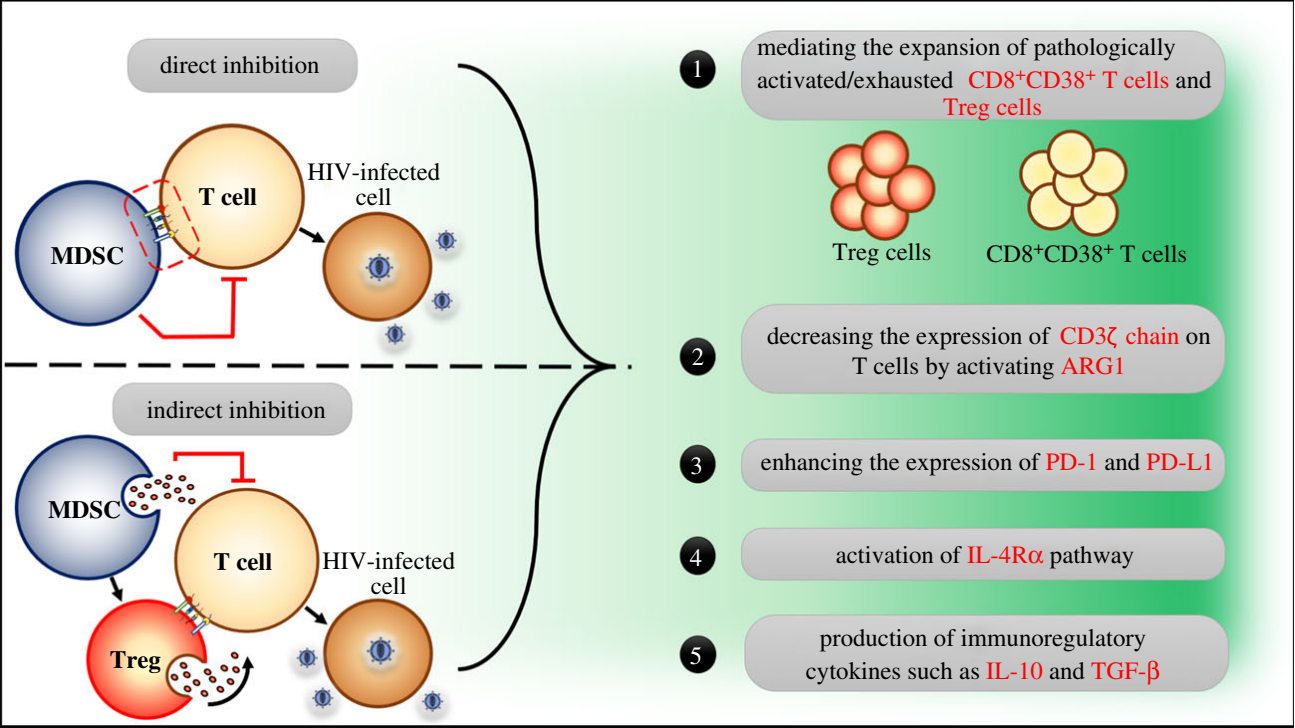
MDSCs inhibit anti-HIV immune responses mediated by T cells. MDSCs could inhibit T cell responses against HIV-infected cells via direct cell-to-cell engagement. Alternatively, MDSCs could inhibit T cells indirectly via secreting anti-inflammatory molecules such as interleukin-10 (IL-10) and transforming-growth factor beta (TGF-β), or via mediating the expansion of other immunoregulatory cells such as regulatory T (Treg) cells. As the case with MDSCs, Treg cells could engage with T cells directly or secrete immunoregulatory molecules to suppress T cell responses against HIV-infected cells. Indeed, MDSCs could directly or indirectly engage with T cells and suppress anti-HIV immune responses mediated by T cells via one or more of the following mechanisms: (1) mediating the expansion of pathologically activated CD8^+^CD38^+^ T cells and or Treg cells; (2) activating arginase-1 (ARG1), which is known to decrease the expression of CD3ζ chain on T cells that, in turn, limits the activation of T cells; (3) enhancing the expression of immunoregulatory checkpoint proteins, namely programmed death cell protein 1 (PD-1) and its ligand (PD-L1); (4) activating interleukin-4 receptor alpha (IL-4R*α*) pathway; (5) production of immunosuppressor molecules such as IL-10 and tumour-growth factor *β* (TGF-β).

**Figure 4.**
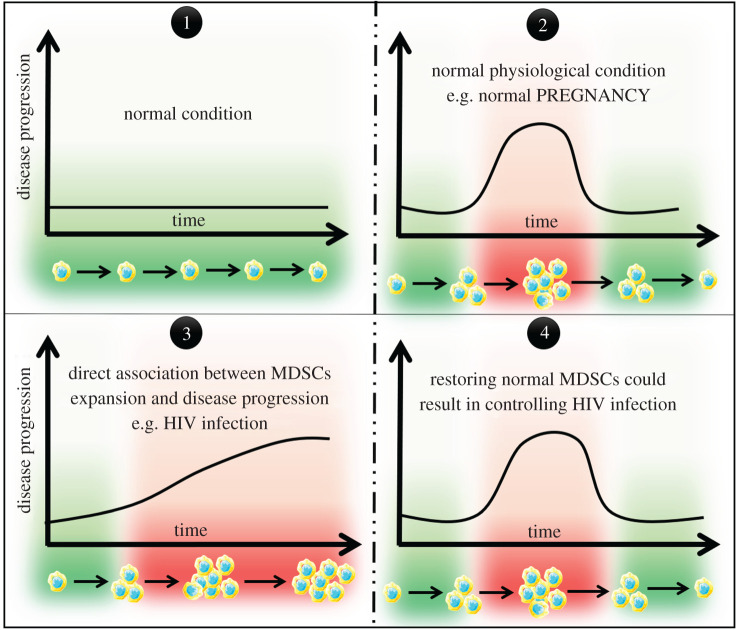
MDSC expansion in health and HIV infection. Under normal physiological conditions, in the absence of inflammation, MDSCs remain at low levels as seen in case number 1. In other normal physiological conditions such as pregnancy and lactation [7], the levels of MDSCs are increased to protect the fetus from its mothers' immune system. After delivery, the levels of MDSCs return to the level before pregnancy as seen in case number 2. In the pathological settings such as HIV infection, as seen in case number 3, there is a direct association between MDSC expansion and HIV disease progression. Therefore, restoring normal MDSC levels, therapeutically, could result in control of HIV disease progression, as seen in the proposed case number 4.

Taken together, it is clearly evident that MDSCs are expanded during early and late HIV infection stages, and such expansion is associated with viral persistence and disease progression [29–35,38,39]. This expansion is driven by different factors including host (such as IFN-α, MCP-1 and MIP-1*α*/*β*), viral (i.e. HIV gp120, Nef and Tat proteins) and microbial (i.e. LPS) factors (figure 2). Expansion of MDSCs during HIV infection plays an indispensable role in the pathogenesis of HIV infection, at least in part, through enhancing HIV replication while suppressing anti-HIV immune responses mediated by T cells in direct and/or indirect manners (figure 1). The latter is accomplished by different mechanisms including: (i) mediating the expansion of potent immunosuppressor cell populations including Tregs [29,34,35]; (ii) mediating the expansion of abnormally activated/exhausted CD8^+^CD38^+^ T-cell populations [30,33]; (iii) downregulating the expression of CD3ζ chain, which is a part of TCR, on CD4^+^, CD8^+^, Vγ9 Vδ2T cells [31,32]; (iv) enhancing the expression of immune checkpoint proteins including programmed cell death protein 1 (PD-1) and its ligand (PD-L1) [31,33,35]; (v); activation of IL-4R*α* pathway [29,35]; (vi) induction of ARG1 [30,83]; and (vii) production of potent immunosuppressive cytokines including IL-10 [33–35] (figure 3). Therefore, targeting MDSCs could result in restoring T responses against HIV and thus controlling HIV disease progression (figure 4).

### 3.3. Myeloid-derived suppressor cells expansion during simian immunodeficiency virus infection

To address the kinetics of MDSCs during SIV infection in rhesus macaques, a non-human primate model of HIV infection was recently used by Dross *et al*. [40]. One major finding of this study was that MDSCs are expanded during the early phase of SIV infection and such expansion continues to the chronic phase of infection. The levels of MDSCs, particularly PMN-MDSCs, were shown to be significantly increased post-SIV infection when compared to those measured before SIV infection. Importantly, such expansion was shown to be directly associated with viral load set-point, a critical marker of disease progression [84]. However, they observed no significant correlation between MDSC expansion and viral load or CD4^+^ T-cell counts. This contradicts the results of human studies in that the viral load in ART-naive chronically infected HIV patients correlates with MDSC frequencies [29–31]. In part, this may be due to the fact that SIV-infected rhesus macaques used in the study were mostly aviremic, unlike ART-naive chronically infected HIV patients which were viremic, since rhesus macaques were treated with ART as early as six weeks post-SIV infection. This indicates that prolonged exposure to viremia in ART-naive chronically infected HIV patients may act as a critical factor that influences the elevation of inflammatory cytokines and thus the induction of MDSC generation and accumulation. On the other hand, the absence of correlation between CD4^+^ T-cell counts and MDSC frequency in the study could be due to the elevation in CD4^+^ T-cell counts in SIV-infected animals (average of 1150 CD4 per ml) versus that in human studies (average of greater than 500 CD4 per ml) [29–31,40]. This is especially true since a significant correlation between MDSC frequency and CD4^+^ T-cell counts may wane in healthy individuals and HIV-infected humans or SIV-infected animals with high CD4^+^ T-cell counts [29–31,40]. To determine the impact of ART on MDSCs, Dross *et al*. have shown that the levels of MDSCs remained significantly high after 31 weeks of ART initiation. Cessation of ART resulted in a remarkable increase in MDSCs and such increase remained after 30 weeks post-ART cessation. Yet, the major purpose of this study was to investigate the connection between MDSC expansion and reduced T-cell proliferation capacity during SIV infection, especially since the proliferative capacity of T cells is considered to be the strongest independent predictor factor of disease progression in HIV patients [85]. Unsurprisingly, similar to what has already been established in human investigations, MDSCs obtained from SIV-infected rhesus macaques were able to suppress T-cell functions and reduce their proliferative capacity (as measured by the expression of CD3 and Ki67) at different time points (3 weeks pre-SIV infection, 3 weeks post-SIV infection, 31 weeks post-ART treatment and 19 weeks post-ART interruption) [40]. With respect to the distribution of MDSCs in peripheral lymphatic tissues or the so-called secondary lymphoid organs (lymph nodes and spleen), which are the sites where T cells become activated, there was no increase in MDSC frequency in the lymph nodes but not the spleen, when compared to the peripheral blood. Additionally, MDSCs from the spleen of SIV-infected rhesus macaques were shown to be as suppressive as the MDSCs of peripheral blood. It is worth mentioning that the splenic infiltrated MDSCs of tumour-bearing mice are less suppressive than those obtained from the tumour site [86]. This seems to be a remarkable difference between retroviral infection and cancer. In the context of determining the force that drives the expansion of MDSCs during the course of SIV infection, Dross *et al*. [40] have shown that unlike viral load, inflammatory cytokines (such as TNF*α*, IL-1*β*, IL-6 and MIP-1*α*/*β*) were the major drivers of MDSC expansion over the viral particles. Generally speaking, it has been shown that the frequency of MDSCs increases as the disease progresses in SIV-infected rhesus macaques, suggesting that MDSCs play a critical role in the pathogenesis of SIV infection [40].

## 4. Myeloid-derived suppressor cells as a therapeutic target in human immunodeficiency virus infection

It is clearly evident that the expansion of MDSCs during the course of HIV infection has detrimental impacts on HIV persistence and disease progression, at least in part, through hampering anti-HIV immune responses mediated by T cells [29–35,83,87]. Also, MDSCs per se could stand as a barrier to reconstitute immune responses in successfully antiretroviral treated HIV patients [35]. Accordingly, targeting MDSCs could provide a promising avenue to enhance and reconstitute anti-HIV immune responses, both of which can participate in controlling HIV infection (figure 4). This is true, especially, if we took into consideration that the expansion of MDSCs accompanies HIV infection from the early days after infection to the late AIDS phase [30,33,37–39]. In general, targeting MDSCs could be achieved by: first, targeting MDSCs for elimination; second, targeting the suppressive function of MDSCs; and/or third, targeting their recruitment to the site of inflammation. The first one could be achieved by targeting factors that regulate their expansion which include viral (gp120, Tat and Nef proteins), host (e.g. inflammatory cytokines, regulatory molecules and transcription factors among others) and microbial (e.g. LPS) factors [30,34,35,83,88–90]. The second one could be achieved for example, by targeting immune checkpoint proteins (PD-1/PD-L1), immunoregulatory cytokines and/or other molecules. The third therapeutic approach could harness, for example, certain chemokine inhibitors to prevent the recruitment of MDSCs to the site of active HIV replication such as the lymphatic tissues. Unfortunately, there are no clinical trials that assess any of the previously mentioned strategies in the context of HIV infection. However, in cancer settings, different classes of drugs that target MDSCs have been used in clinical trials. With respect to anti-cancer candidate drugs that target MDSCs for elimination, for example, all-trans retinoic acid, certain tyrosine kinase inhibitors and TRAIL-R2 agonists were successfully used to eliminate MDSCs in cancer patients [91–96]. With respect to targeting the suppressive function of MDSCs, using certain phosphodiesterase inhibitors [97,98] and STAT3 inhibitors [99] has been reported to reduce the suppressive activity of MDSCs in cancer patients. Finally, different classes of drugs belong to CCR5 antagonists [100], S100 protein antagonists [101], CCL2 inhibitors [22] and VEGF inhibitors [102] were shown to block the recruitment of MDSCs to the site of inflammation as a strategy to limit the inhibitory effect of MDSCs in cancer tissues. The promising results obtained from targeting MDSCs in cancer settings at the clinical level suggest that such therapeutic approaches/drugs could be used in the context of HIV infection in the future.

## 5. Conclusion

Taken together, there is no doubt that MDSCs are expanded during the acute and chronic phases of HIV and SIV infections in humans and rhesus macaques, respectively. Such expansion is regulated by different factors, including host, viral and microbial factors (figures 1 and 2). Importantly, the expansion of MDSCs during HIV infection is associated with disease progression. This work aimed to address the mechanisms by which MDSC expansion contributes to the pathogenesis of HIV infection. As such, we included all the published studies related to the pathological role played by MDSCs during HIV infection to the date of paper submission. Regarding the mechanisms by which MDSCs sabotage anti-HIV immune responses in these studies, great variation existed in the results, although they were consistent with the findings observed in other pathological conditions. In other words, under certain circumstances, MDSCs induce their immunosuppressive activity directly upon cell-to-cell engagement through PD-1/PD-L1-axis, or indirectly by secreting immunosuppressant molecules such as IL-10, activating or mediating the expansion of Treg cells and exhausted CD8^+^CD38^+^ T cells, or even through a combined event (figure 3). Activation of IL-4R*α* pathway and ARG1 were also reported. Indeed, the activation of ARG1 can reduce the expression of CD3ζ chain, which is a part of the TCR, on T cells, thereby, reducing the activation of T cells upon antigen stimulation. However, the research field on MDSCs during HIV infection is still in its infancy because of the following reasons: (i) the expansion of MDSCs is still undetermined in anatomical compartments other than peripheral blood [103]; (ii) the suppressive activity of MDSCs has been investigated only in T cells but not in other immune cells such as DCs, monocytes/macrophages and NK cells; (iii) there are no investigations about the suppressive activities of MDSCs in HIV-infected elite controllers (a group of patients that control HIV replication to a level below the limit of detection by standard assays); and (iv) targeting MDSCs therapeutically has neither been investigated in pre-clinical nor in clinical sides during HIV infection. Therefore, future investigations should address these issues to fill the gap of knowledge in this important area of research.

## Data accessibility

This article has no additional data.
